# Retraction Note: Psoralen synergizes with exosome-loaded SPC25 to alleviate senescence of nucleus pulposus cells in intervertebral disc degeneration

**DOI:** 10.1186/s13018-026-06826-z

**Published:** 2026-03-31

**Authors:** Lei Yang, Zhaoyong Li, Chao Zhang, Shuofu Li, Long Chen, Shaofeng Yang, Yantao Guo

**Affiliations:** Department of Spine, The First Affiliated Hospital of Hunan University of Traditional Chinese Medicine, No. 95 Shaoshan Middle Road, Yuhua District, Changsha, 410007 Hunan People’s Republic of China

**Retraction Note to: Journal of Orthopaedic Surgery and Research (2023) 18:622** 10.1186/s13018-023-04085-w

The Editor-in-Chief has retracted this article at the request of the authors. After publication, the authors informed the Journal that the experiments reported in this publication were conducted by a third-party laboratory. This is in contravention of the Journal’s Editorial Policies, which state that when data analysed or generated by a third party, this should be clearly stated within the article’s Methods and/or Availability of data and Materials sections. The authors also informed the Journal that they had lost confidence in the integrity of the data they had been provided with. The Editor-in-Chief, therefore, no longer has confidence in the research presented in this work.

All authors agree with this retraction.

